# Intravesical Injection of Botulinum Toxin Type A in Men without Bladder Outlet Obstruction and Post-Deobstructive Prostate Surgery

**DOI:** 10.3390/toxins15030221

**Published:** 2023-03-15

**Authors:** Hsiang-Ying Lee, Hann-Chorng Kuo

**Affiliations:** 1 Department of Urology, Kaohsiung Medical University Hospital, Kaohsiung 80756, Taiwan; ashum1009@hotmail.com; 2 Department of Urology, School of Medicine, College of Medicine, Kaohsiung Medical University, Kaohsiung 80756, Taiwan; 3 Graduate Institute of Clinical Medicine, College of Medicine, Kaohsiung Medical University, Kaohsiung 80756, Taiwan; 4 Center for Cancer Research, Kaohsiung Medical University, Kaohsiung 80756, Taiwan; 5 Department of Urology, School of Medicine, Tzu Chi University, Hualien 970473, Taiwan; 6 Department of Urology, Hualien Tzu Chi Hospital, Buddhist Tzu Chi Medical Foundation, Hualien 970473, Taiwan

**Keywords:** botulinum toxin, overactive bladder, men

## Abstract

Purpose: A significant proportion of men without bladder outlet obstruction (BOO) have been reported to have overactive bladders (OAB). This article aimed to review the specific group of reports on the use of botulinum toxin type A (BTX-A) injections into the bladder wall. Materials and methods: Original articles reporting men with small prostates without BOO were identified through a literature search using the PubMed and EMBASE databases. Finally, we included 18 articles that reviewed the efficacy and adverse effects of BTX-A injections in men. Results: Of the 18 articles screened, 13 demonstrated the therapeutic efficacy and adverse effects of BTX-A injections in men. Three studies compared BTX-A injection response between patients without prior prostate surgery and those undergoing prior prostate surgery, including transurethral resection of the prostate and radical prostatectomy (RP). Patients with prior RP experienced better efficacy and had a low risk of side effects. Two studies focused on patients who had undergone prior surgery for stress urinary incontinence, including male sling and artificial urethral sphincter surgery. The BTX-A injection was a safe and effective procedure for this specific group. OAB in men was found to have a different pathophysiology mechanism from that in female patients, which may decrease the efficacy of BTX-A injection in men. However, patients with small prostates and low prostate-specific antigen levels demonstrated better efficacy and tolerability after BTX-A injection. Conclusions: Although intravesical injection of BTX-A was a good option for controlling refractory OAB in men, the evidence-based guidelines are still limited. Further research is necessary to better understand the role of BTX-A injections on various aspects and histories. Therefore, treating patients using strategies tailored to their individual conditions is important.

## 1. Introduction

Lower urinary tract symptoms (LUTS) are common in male patients with bladder outlet obstruction (BOO) [1]. Subsequent detrusor physiological alterations, including hypertrophy, denervation caused by ischemia, and changes in neuronal mechanisms resulting from obstruction may lead to overactive bladder (OAB) [2]. OAB is a bothersome condition defined as a symptom complex of urinary urgency. Usually, it is accompanied by frequent urination, nocturia, and urinary incontinence. The prevalence of OAB in men has been reported to be approximately 26–33% in the United States, which increases with age, such that 73.9% of men over 60 years report urinary storage symptoms [3,4]. In the EPIC study, 10.8% of the male population were shown to have OAB [5]. However, a significant proportion of male individuals had OAB symptoms even without obstruction. In a total of 128 young men who underwent a urodynamic study (UDS) by Manohar et al., 18% had OAB without BOO [6].

Intervention for OAB in male patients is similar to that in female patients, usually beginning with behavioral treatment and medication management, including antimuscarinic agents and β3 agonists. However, in patients with persistent OAB symptoms despite conservative and medication treatment, defined as refractory OAB, more aggressive and invasive interventions are required, including intravesical injection of onabotulinum toxin A (BTX-A). BTX-A injections were approved by the US Food and Drug Administration for treatment in patients with non-neurogenic OAB in 2013 [7]. Most previous studies have evaluated the efficacy and adverse effects of BTX-A injections in female patients. However, there is a paucity of data on male patients with OAB. In a study by Rahnama’I et al., the success rate of intravesical BTX-A injection in men was 21%, with a mean follow-up of approximately 69 months. The most common reasons for discontinuing BTX-A injections were insufficient efficacy and side effects [8]. A recent systematic review that discussed BTX-A injections in men with OAB concluded that BTX-A injections could induce therapeutic response and have an impact on urodynamic parameters. Although the review article enrolled <1000 men, the available evidence was heterogeneous and limited [9].

The pathophysiology of OAB with or without BOO may differ. This review focuses on BTX-A injections in men with a small prostate size or history of prostate surgery. Studying the effect of BTX-A in this subgroup of patients would help clinicians better understand the response and adverse effects, and whether patients with small prostates were more likely to respond favorably to BTX-A injection compared to those with obstructed urethra due to large prostates.

## 2. Evidence Acquisition

A literature search of PubMed and EMBASE databases was conducted in October 2022, screening all topics on intravesical BTX-A injection in male patients without BOO who had refractory OAB after medication treatment. The search strategy included the following keywords/mesh terms: “Botox injection” and “overactive bladder”. The searches were pooled with the limitations of men and language (English). Thereafter, animal model studies and review articles were excluded. Children and patients with neurogenic bladder were excluded from the study. Congress abstracts and book chapters were not considered for discussion in this review article.

**Figure 1:**
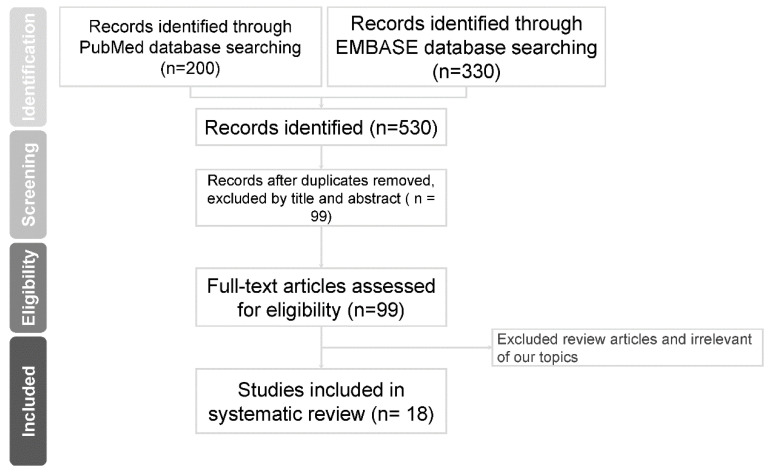
Flowchart of included articles for review.

After removing duplicates, we screened titles and abstracts to select appropriate studies and exclude unrelated articles. Initially, 99 related original articles retrieved from PubMed and EMBASE were included. Additionally, full-text articles were further assessed. Many studies have included both sexes but have not evaluated them separately. To focus on the male population, we only used studies in which data could be identified as results from men without BOO. We finally included 18 articles for our narrative review. Most of the articles were retrospective, single-center studies. A flowchart of this process is shown in Figure 1.

## 3. Therapeutic Effectiveness and Adverse Events after BTX-A Injection

BTX-A intravesical injection focusing on male patients without BOO is presented in Table 1.

Regarding the mechanism underlying BTX-A, it inhibits signal transmission at the neuromuscular junction by inhibiting acetylcholine release, which interferes with the binding of neurotransmitters to postsynaptic receptors. Wang et al. [10], in Taiwan, reported a post-marketing survey of intradetrusor BTX-A injections in patients with OAB, including 62 male individuals. There was a significant improvement in OAB symptoms according to the Patient Perception of Bladder Condition and OAB Symptom Score questionnaires at 4 and 12 weeks after intervention compared with baseline. In terms of adverse events, although increased post-voiding residual urine (PVR) was found at 4 and 12 weeks, the PVR at 12 weeks declined compared with that at 4 weeks. Moreover, there were no patients with de novo acute urine retention (AUR). The incidence of urinary tract infection (UTI) was low (4.6%). Acceptance of BTX-A injections among patients was high owing to its acceptable efficacy and safety. BTX-A has been shown to be effective in patients with and without urinary incontinence. Grishin et al. [11] divided enrolled patients, including men and women, into OAB without imperative urinary incontinence (Group 1) and OAB with imperative urinary incontinence (Group 2). After 200 units of BTX-A injection, a decrease in the number of urinary incontinence episodes by 1.59 times (*p* < 0.05) in Group 1 and 2.75 times (*p* < 0.05) in Group 2 was found. The quality of life (QoL) also improved according to the SF-36.

The pathophysiology of OAB has been suggested to differ between men with and without BOO. Regarding antimuscarinic treatment, previous research demonstrated that patients with OAB with smaller prostates might benefit from antimuscarinic agents alone without adding σ-adrenergic agents to relieve OAB symptoms. Men with OAB due to primary bladder conditions may respond well to antimuscarinic therapy alone [12]. A positive correlation between prostate-specific antigen (PSA) levels and prostate size was confirmed before high PSA levels could be translated to a large prostate volume [13]. Similarly, Roehrborn et al. demonstrated that among patients with PSA level < 1.3 ng/mL, tolterodine ER alone significantly improved the frequency and International Prostate Symptom Score storage scores compared with placebo [14]. Theoretically, patients with OAB with a small prostate size would likely benefit from intravesical BTX-A injections. In a phase III randomized controlled phase (RCT) study, Yokoyama et al. [15] compared BTX-A injection and placebo groups, dividing participants into lower PSA levels (<1.5 ng/mL) and higher PSA levels (≥1.5 ng/mL). The cut-off value was according to previous research in which PSA ≥ 1.5 ng/mL could be considered an enlarged prostate (>30 mL) [16]. BTX-A injection was effective, with tolerable adverse effects in patients with lower PSA levels. Urinary incontinence episodes showed a greater decline compared with the placebo in patients with small prostates. However, no significant improvement in OAB symptoms was observed in the subgroup with higher PSA levels. The risk of urine retention in patients with lower PSA levels was low, owing to a small elevated PVR.

Men with higher PSA levels were considered to have larger prostate volume, which might worsen OAB symptoms due to BOO [17]. Abrar et al. [18] demonstrated that male sex was a significant predictor of poor response in patients with LUTS and associated with a higher risk of clean intermittent self-catheterization (CISC) after BTX-A injection compared with female sex. Although men with BOO were excluded from this study, prostate enlargement may still be a possible reason why OAB symptoms might partially result from voiding resistance. However, the treatment response rate in men was 62% in a study conducted by Mateu Arrom et al. [19], which showed that the efficacy of BTX-A injection was similar to that in the female population. Based on urodynamic results, in terms of objective outcome evaluation, decreased detrusor pressure, maximum flow rate, and impaired voiding efficiency were observed 3 months after injection. In this study, 13% of the patients required CISC, which may be slightly higher than that in female patients. Although male patients without BOO may experience similar benefits to those in the female population after receiving Botox injections, the extent of prostate urethral resistance is still the main reason for the worsening efficacy, with a higher risk of complications. The authors observed that the BOO index (BOOI) was related to the BTX-A injection response and development of complications after treatment. Careful evaluation of BOOI before BTX-A intervention may be useful for predicting efficacy and adverse effects. Since only 30–50% of patients with OAB could have detrusor overactivity (DO) [20], Kanagarajah et al. [21] included only patients with OAB without DO. Both sexes showed similar improvements in the urogenital distress inventory-6 questionnaire and visual analog scale scores at week 12 post-injection. A study by Walker et al. [22] indicated that some men developed de novo CISC after receiving a BTX-A injection, even with BOOI < 20. However, according to the results of a questionnaire in a real-life clinical setting in men, BTX-A injection could objectively improve QoL. Patients with good detrusor function in terms of bladder contractility index value > 150 were less likely to require de novo CISC.

Hsiao et al. [23] evaluated the factors affecting therapeutic efficacy after BTX-A injection. They found that symptoms could improve six months after injection in both sexes. However, male sex was associated with worse therapeutic efficacy compared with female sex. The success rate was 63.8% (male and female). In a further study by the same research group [24], they assessed if the urodynamic factors could predict large PVR urine volume after BTX-A injection. A total of 44% of male patients experienced large PVR (>200 mL) during follow-up. Daytime frequency episodes and voiding efficiency were significant predictors of a large PVR. They discovered that sex was not one of the causative factors, and both sexes may have induced large PVR during the 6-month follow-up period. However, male patients showed a shorter persistent PVR (>150 mL) interval than did women in terms of faster recovery from a large PVR.

The adverse effects of BTX-A injections were discussed in a study by Jiang et al. [25]. Male patients (14.2%) experienced AUR and 28.4% had large PVR (≥200 mL) in one month. The initial greater increase in PVR in male patients than in female ones was not significant after one month. UTIs developed in 8.8% of male patients. The incidence of AUR was significantly higher in male patients than in female ones. However, female patients had a higher incidence of UTI compared with male patients. Similarly, Kuo et al. [26] revealed that male patients had a higher risk of AUR after BTX-A injection using both univariate and multivariate analyses and a higher incidence of UTI adverse events than female patients. When selecting male patients with OAB to receive BTX-A injections, even without BOO, the possibility of AUR should be considered. However, the success rate in male patients was 67%, which was not significantly different from that in female patients (66%). Specifically, male patients with a history of prior transurethral resection of the prostate (TURP) had a higher success rate (74%) compared with no TURP history cohort, without a statistically significant difference. Male patients without TURP history had higher incidence of hematuria and UTI, which may have resulted from prostate-related adverse effects. According to baseline UDS variables, detrusor pressure during voiding was not related to adverse event occurrence after BTX-A injection in male patients. In contrast, Liao et al. [27] evaluated the impact of BTX-A injections on refractory idiopathic detrusor overactivity. They found that the success rate at 12 months in frail older patients was lower than that in older patients without frailty and younger patients (6.82%, 22.3%, and 23.1%, respectively). Sex was not a factor inducing a higher risk of PVR > 150 mL. However, frail older patients were at greater risk for a large PVR and a higher chance of requiring an indwelling catheter or CISC. Similarly, in a study by Osborn et al. [28], male patients did not have an increased risk of urinary retention after BTX-A injections. Postoperative urinary retention was defined as the requirement for a CISC or indwelling catheter. Therefore, the threshold postoperative PVR urine volume to initiate CISC could determine postoperative urinary retention rate.

Patients with refractory OAB required additional BTX-A injections for two reasons. One reason was the lack of medication efficacy. The other reason was the intolerable adverse effects of anticholinergic agents. Makovey et al. [29] reported that more men had a history of anticholinergic medication efficacy than of intolerable side effects. They further indicated that BTX-A injection had a higher success rate in patients with refractory idiopathic OAB due to anticholinergic intolerability than in those with poor medication efficacy.

## 4. The Impact of Injection Site, Dosage, and Numbers of BTX-A

Previous studies have assessed whether the injection site can affect the efficacy and adverse effects of BTX-A injections. The current guidelines recommend that BTX-A should not be injected over the trigone area, based on the results of early research that exhibited a potential risk of development of vesicoureteral reflux after trigone area injections [30]. However, further evidence did not support this risk. El-Hefnawy et al. [31] compared trigonal-sparing versus trigonal-involved BTX-A injections and found that improvement in all components of OAB symptoms occurred at both sites. Additionally, the response reached maximum efficacy in the third month. Significant improvements in episodes of urge incontinence and urinary frequency were observed in the trigonal-sparing group at six months. In contrast, higher UTI incidence rate and detrusor leak point pressure were found in the trigonal-involved injection group. The abundance of sensory fibers in the trigonal area, which is considered to play a role in bladder urgency sensation, is assumed to induce a better response after BTX-A injection. Kuo et al. [32] demonstrated that intravesical BTX-A injection was an effective treatment, regardless of the injection site. In addition, detrusor and suburothelial injection techniques showed similar efficacies in idiopathic detrusor overactivity (IDO). Various physiological mechanisms are related to urinary function within the suburothelial space, including sensory and solute transport. The effect of BTX-A injection generally diffuses from the detrusor muscle and suburothelial space [33]. The function of detrusor injection may be due to an effect on acetylcholine release in the neuromuscular junction. In contrast, suburothelial injection might affect the sensory receptor, which further mediates detrusor contractions. When performing BTX-A injections over the detrusor muscle, one should be cautious as BTX-A may be lost outside the bladder if the needle passes over the bladder wall. Suburothelial injection can retain BTX-A within the suburothelium and it is easier to visualize the mucosal swelling, which has more precise toxin localization, if the injection site is appropriate.

Regarding idiopathic OAB, 100 units of intravesical BTX-A is recommended. Abdelwahab et al. [34] compared between 100- and 200-unit BTX-A intradetrusor injections. They revealed that the efficacy was similar regardless of the BTX-A dosage, and a higher incidence of adverse effects occurred in patients receiving 200 units of BTX-A after nine months of follow-up. Therefore, an injection of 100 units could be sufficient to achieve satisfactory outcomes in patients with idiopathic OAB with or without IDO [35]. In addition to the dosage of BTX-A, the number of injection sites did not affect treatment outcomes. A prospective randomized study compared patients receiving different numbers of intravesical 100-unit BTX-A injections in 10 mL (10, 20, and 40) into the bladder body. It has been demonstrated that 10 sites were adequate to achieve equal therapeutic effects to 20 and 40 sites [36]. The number of injections was less relevant by spreading the BTX-A solution across the suburothelial space. BTX-A injection is performed as an office-based procedure under local anesthesia. Therefore, a small number of injection sites might reduce adverse effects and uncomfortable experiences due to injection, such as bladder pain, hematuria, and possible UTI [36].

Repeated injections of BTX-A were as efficacious as the first injection, without resistance. The results showed improvements in OAB symptoms, urodynamic parameters, and QoL. The most common reason for dropping out was poor response or dislike of CISC. The interval between different injections was approximately 9–10 months. Additional evidence would eliminate the fear of toxin accumulation with repeated injections [37,38].

## 5. BTX-A Injection after Deobstructive Prostate Surgery

Intravesical BTX-A injection is a treatment option for relieving obstruction in men with persistent OAB after prostate surgery. However, studies involving male patients are limited. From a histopathological perspective, persistent DO following TURP may result from the increased resistance of bladder vessels and decreased perfusion [39]. Whether the histopathology differs between men who have undergone prostate surgery and those who have not remains unclear.

Habashy et al. [40] enrolled male patients who underwent prior prostate surgery, including radical prostatectomy (RP) and TURP, without undergoing surgery. Comparing prostate surgery with no surgery groups showed significant improvement in pad usage after BTX-A injection. However, there was no significant difference between the two groups in Patient Global Impression of Improvement (PGI-I) scores. In the subgroup analysis of patients who underwent prostate surgery, the RP group showed greater improvements in pad usage and PGI-I scores than did the TURP group. The reason for the different BTX-A injection efficacies is unclear. It is suggested that the TURP group had more severe detrusor dysfunction due to BOO, while the RP group had a relatively higher degree of sphincteric deficiency, which is the main reason for incontinence.

The long-term efficacy of BTX-A injections was demonstrated by Bels et al. [41] in subgroups based on prior prostate surgery. The discontinuation rate was 70.8% during the 23-month median follow-up. TURP, RP, and no-prior-prostate-surgery subgroups after BTX-A injection were compared. The results showed a higher incidence rate of necessary CISC and larger PVR volume in the no-prior-prostate-surgery subgroup than in the prior TURP or RP subgroups. The reason that patients without prior prostate surgery had a higher risk of adverse effects may be that BOO was more prominent in the subgroup without prior prostate surgery (50% of the enrolled patients). The RP subgroup had the lowest CISC rates in terms of a lower discontinuation rate with more tolerable BTX-A injections. This highlights the importance of evaluating the degree of obstruction and prostate size before BTX-A injection to minimize the possibility of de novo CISC.

Another study by Rahnama’I et al. [8] evaluated the long-term compliance and side effects of BTX-A injections in heterogeneous groups of male patients. The success rates of idiopathic OAB, TURP, and post-PCa treatments after a mean follow-up period of 69 months were 21%, 11%, and 29%, respectively. Similar to the studies by Habashy et al. and Bels et al. [39,40], patients with prior RP had better efficacy and a lower risk of side effects compared with patients with prior TURP. The insufficient effect of poor satisfaction after BTX-A injection in the post-TURP subgroup may be due to moderate detrusor dysfunction resulting from a history of long-term obstructed prostate.

## 6. BTX-A Injection after Stress Urinary Incontinency Surgery

Artificial urinary sphincters (AUS) and male slings are popular treatments for post-RP stress urinary incontinence (SUI). Preoperative OAB symptoms may persist after SUI surgery and 23–37.5% of patients develop de novo OAB after AUS implantation [42]. BTX-A injection is still considered an option for controlling refractory OAB symptoms after treatment. De Sallmard et al. showed that a significant number of male patients (90%) with an AUS implantation history had totally or partially improved OAB symptoms after injection. The discontinuation-free survival rate was 50% at 60 months. However, this was combined with the female results. Nevertheless, this study indicated that BTX-A injections were effective in patients undergoing AUS implantation. No significant BTX-A-related adverse effects were encountered after injection: two male patients experienced urethral erosion, which may have resulted from the temporarily necessary CISC. Therefore, the incidence of CISC may be related to the risk of AUS complications.

Mateu Arrom et al.’s study [43] enrolled patients with prior RP and TURP and a history of SUI surgery, including AUS and male sling implantation. At a median follow-up of 49 months, 66.7% of the patients showed subjective improvement in OAB symptoms after injection. All patients were confirmed to have DO before BTX-A injection using the UDS. There was a significant reduction in the proportion of patients with DO after BTX-A injections (from 100% to 53.3%). According to the UDS data, no significant change in voiding efficiency or PVR was noted three months after treatment. The results indicated a good response to treatment and a low complication rate in patients with prior SUI surgery. The authors mentioned that the procedure should be performed with caution to prevent urethral injury, which may increase the risk of further AUS cuff erosion. They recommended avoiding injection of BTX-A over the balloon side in cases of balloon perforation.

## 7. Conclusions

The available evidence regarding BTX-A injection for refractory OAB in male patients is limited. Furthermore, most studies have combined BTX-A injection in female patients to analyze the efficacy or adverse effects of BTX-A injection. Generally, the response rate in male patients was similar to that in female patients. However, some studies have indicated that female patients exhibited better therapeutic efficacy, considering that prostate-related OAB has a different pathophysiology. However, owing to the distinct pathophysiology between men with and without BOO, patients with a small prostate and without BOO or a history of prostate surgery could have a better response and fewer adverse effects after BTX-A injection. The degree of outlet resistance may determine the efficacy of BTX-A injections in OAB symptom control. Compared with prior prostate surgeries, patients receiving RP showed better improvement in OAB symptoms and the lowest CISC rates after BTX-A injection.

Male sling and AUS were surgical choices in patients with SUI after RP. Some patients showed persistent or de novo OAB symptoms after SUI surgery. It might be feasible for patients with prior SUI surgery to receive BTX-A injections, which could improve DO and was safe. However, caution should be observed about the possible complications, despite their rarity, such as balloon perforation and urethral erosion.

## Figures and Tables

**Table 1:** Therapeutic effectiveness and adverse events after BTX-A injection.

Study	Design	Population Number of Men	Dose/Units	Technique	Comparator	Outcomes	Follow-Up Schedule
Wang et al. [10]	Prospective cohort study	62 men Treatment-naïve to Botox injection	100 units	Injection into the detrusor at 10 points	NA	Improving PPBC and OABSS at 4 and 12 weeks	1 week 4 weeks 12 weeks
Yokoyama et al. [15]	RCT, phase III	62 men Patients with BOO were excluded	100 units	Injection into the detrusor at 20 points	BTX-A vs. Placebo	Greater decrease in UI episodes in men with lower PSA levels	2 weeks 6 weeks 12 weeks
Abrar M et al. [18]	Retrospective cohort study	24 men	10 units/mL/injection site	NA	NA	Men had worse responses and required CISC more than women	4–6 weeks
Mateu Arrom L et al. [19]	Retrospective cohort study	146 men	100 units	20 points excluding the trigone	Pretreatment vs. posttreatment	62% response rate	3 months
Kanagarajah P et al. [21]	Prospective cohort study	5 men	100 or 150 units	10 points	Baseline UDI-6 and VAS score vs. postinjection UDI-6 and VAS score	Both genders showed improvements in UDI-6 and VAS score in patients without DOA	12 weeks
Faure Walker NA et al. [22]	Retrospective comparative study	65 men	Initial 200 → start at 100 units	10–20 points	Men vs. women	Improving QoL	4–12 weeks
Hsiao SM et al. [23]	Retrospective cohort study	46 men	100 units	20 points excluding the trigone	Men vs. women	Male gender was associated with worse therapeutic efficacy	6 months
Hsiao SM et al. [24]	Retrospective cohort study	148 men	100 units	20 points excluding the trigone	NA	Men had shorter persistent PVR > 150 mL intervals than women	6 months
Jiang YH et al. [25]	Retrospective cohort study	148 men	100 units	20–40 points excluding the trigone	AUR vs. large PVR vs. UTI	Men had more AUR postinjection than women	6 months
Kuo HC et al. [26]	Prospective cohort study	112 men	100–200 units	20 points including the trigone	NA	Men had more AUR postinjection than women	12 months
Liao CH et al. [27]	Retrospective cohort study	93 men	100 units	40 points excluding the trigone	Frail elderly vs. elderly without frailty vs. people younger than 65 years	Men did not have an increased risk of PVR greater than 150 mL	12 months
Osborn DJ et al. [28]	Retrospective cohort study	38 men	100 or 200 units	NA	No retention vs. retention	Gender was not associated with postoperative urinary retention	40 weeks
Makovey I et al. [29]	Retrospective cohort study	17 men	150–200 units	20 points including the trigone	Lack of efficacy vs. intolerable side effects	Lack of anticholinergic efficacy had less success in Botox injections	11 months

NA: Not available.

## Data Availability

Not applicable.
